# An outbreak of monophasic *Salmonella* Typhimurium associated with raw pork sausage and other pork products, Denmark 2018–19

**DOI:** 10.1017/S0950268819002073

**Published:** 2019-12-09

**Authors:** I. G. Helmuth, L. Espenhain, S. Ethelberg, T. Jensen, J. Kjeldgaard, E. Litrup, S. Schjørring, L. Müller

**Affiliations:** 1 European Programme for Intervention Epidemiology Training (EPIET), European Centre for Disease Prevention and Control (ECDC), Stockholm, Sweden; 2 Infectious Disease Epidemiology and Prevention, Statens Serum Institut, Copenhagen, Denmark; 3 Department of Public Health, Faculty of Health and Medical Sciences, University of Copenhagen, Copenhagen, Denmark; 4 Division for Food and Feed Safety, Danish Veterinary and Food Administration, Copenhagen, Denmark; 5 National Food Institute, Technical University of Denmark, Kgs. Lyngby, Denmark; 6 Bacteria, Parasites and Fungi, Statens Serum Institut, Copenhagen, Denmark

**Keywords:** Food-borne infections, outbreaks, salmonellosis

## Abstract

In Denmark, outbreaks of salmonella with more than 20 cases have become rare. In November 2018, an outbreak of monophasic *Salmonella* Typhimurium was detected and an investigation initiated with the aim of identifying the source and controlling the outbreak. Outbreak cases were defined based on core genome multilocus sequence types. We conducted hypothesis-generating interviews, a matched case-control study, food sampling and trace-back investigations. We identified 49 cases distributed across Denmark. In univariable analyses a traditional form of raw Danish pork sausage (medister sausage), pork chops and ground veal/pork showed matched odds ratio of 26 (95% CI 3–207), 4 (95% CI 1–13) and 4 (95% CI 1–10), respectively. In a multivariable analysis, only medister sausage remained significant. Several patients described tasting or eating the sausage raw or undercooked. Samples of medister sausage analysed were negative for salmonella and investigations at the production site did not reveal the mechanism of contamination. In conclusion, in spite of having eliminated salmonella in the egg and broiler industry, Denmark is still at risk of major salmonella outbreaks. We identified a raw pork sausage as a particular risk product that needs to be thoroughly cooked before consumption. Tasting raw meat or eating undercooked pork should be discouraged.

## Introduction

In Denmark, the endemic level of salmonellosis has been markedly reduced over the last two decades. The incidence in 2017 was 18.5/100 000 inhabitants and 50% of cases were infected abroad. The most common serotype in human cases was *Salmonella enterica* subspecies *enterica* serovar Typhimurium, of which the monophasic variant (4,[5],12:i:-) accounted for 60%, followed by the serovar Enteritidis [1, 2]. Due to an effective control programme, *Salmonella* Enteritidis is *de facto* eliminated in the Danish broiler and egg production [3, 4]. *Salmonella* Typhimurium does, however, still exist in Danish pigs and pork where it has the potential to cause outbreaks, although large national foodborne outbreaks with more than 20 cases have become increasingly rare in recent years [1, 5–7].

Since 2017, all isolates from the national surveillance of salmonella in animals, food and humans have been whole genome sequenced (WGS) as part of the surveillance programme. Human cases of salmonellosis are laboratory notifiable and isolates are analysed by WGS at the Statens Serum Institut (SSI). Genetic clusters are initially defined by SSI and the investigation of national outbreaks is coordinated by the Central Outbreak Management Group with participation from SSI, the Danish Veterinary and Food Administration (DVFA) and the National Food Institute at the Technical University of Denmark (DTU-FOOD) [1].

On 16 November 2018, SSI noted eight cases of monophasic *S.* Typhimurium belonging to the same WGS cluster. The specific sequence type (ST) had not been detected before, but was closely related to a ST often found in pork and pork products (ST 34). An outbreak was declared and an investigation initiated with the aim to identify the source and control the outbreak. In the initial eight hypothesis generating interviews, seven cases mentioned that they had eaten a certain type of classical Danish raw pork sausage (medister sausage).

## Methods

### Typing of human isolates

The salmonella isolates were sequences on an Illumina NextSeq platform and run through an in-house QC pipeline (https://github.com/ssi-dk/bifrost). Genome sequences were analysed by cgMLST using the in-house installed calculation engine (BioNumerics, Applied Maths) with the Enterobase scheme (https://enterobase.warwick.ac.uk/species/index/senterica). A genetic cluster was defined based on a single-linkage analysis as two or more closely related genomes with a clear separation from other genomes. Clusters were designated by the 7-locus ST and a consecutive numbering of clusters as they were detected, in this outbreak exemplified by genetic cluster ST5296 cluster 1.

### Case definition

A case was defined as a domestically acquired, laboratory confirmed case of monophasic *S.* Typhimurium belonging to the specific genetic cluster ST5296 cluster 1 with a symptom onset from October 2018–January 2019 in Denmark.

### Hypothesis-generating interviews

In order to describe the outbreak and generate a hypothesis about a possible vehicle of the outbreak, patients were interviewed by telephone using an in-house salmonella specific hypothesis-generating questionnaire.

### Case-control study

A matched case-control study was conducted in order to test the hypothesis that a certain type of classical Danish raw pork sausage (medister sausage) was the vehicle of the outbreak. We included cases with an isolate receipt date at the reference laboratory at the SSI before 5 December 2018. We extracted 10 possible controls per case from the Danish Civil Registration System [8] individually matched on sex, age (birthday ±30 days) and municipality. We aimed to include three controls per case. Cases and controls were excluded if they were not able to complete a telephone interview. Controls were excluded if they had symptoms of gastrointestinal illness or if they had been travelling outside of Denmark a week prior to the interview. A short tailored questionnaire was developed and set up in the web-based survey tool ‘Enalyzer’ (IBM, USA) and cases and controls were interviewed by telephone. The questionnaire included questions on consumption of various types of pork, beef and chicken as well as household shopping sites in the 14 days prior to date of onset of disease (prior to the interview for controls). Cases were also asked about clinical symptoms, duration of illness and hospitalization. Cases that had been interviewed using the hypothesis-generating questionnaire before the case-control study was initiated, were included in the case-control study with the answers they had already provided.

Data exported from Enalyzer was analysed in Stata v. 14 (StataCorp, USA). Univariable conditional logistic regression analyses were performed to assess the relationship between each exposure and disease, calculating matched odds ratios (mOR) and 95% confidence intervals (CI). Exposures that showed a significant positive association with disease were included in a multivariable analysis.

### Analysis of food products

In order to determine the relatedness with the human outbreak isolates, all relevant *S.* Typhimurium isolates (including monophasic variants) sequenced by DVFA in the National salmonella surveillance from animals, food and feed in 2017 (*n* = 187), 2018 (*n* = 315) and 2019 (*n* = 80) were analysed. This was performed by single-nucleotide polymorphisms (SNP) analysis using the SNP pipeline CSI Phylogeny version 1.4 from Center for Genomics Epidemiology, DTU [9]. Furthermore, when interviewed, cases were asked if leftovers of relevant products were available for collection and analysed for salmonella by the DVFA.

### Consumer purchase data investigation and trace-back investigation

In order to investigate whether a specific brand or product batch of medister sausage was responsible for the outbreak, a consumer purchase data investigation [10] was initiated. Cases, who had eaten medister sausage, were contacted and asked if they would provide receipts from supermarket purchases or alternatively data from their web-bank statements (amount spent on a supermarket purchase, credit card number, name of supermarket and date of purchase) in a time period up to 14 days before date of onset of illness or from the specific date they bought medister sausage. This information was then passed on to the relevant supermarkets and food purchase data with information on specific brands of medister sausage was then collected from the supermarket databases.

Trace-back of medister sausage was based on information from the hypothesis-generating interviews, case-control interviews and the consumer purchase data investigation. The trace-back information from cases was compared with the information from the manufacturer concerning the production and marketing of their different medister sausage brands.

Further investigations concerning the production information and own-check results were performed. This included obtaining manufacturers own-check records concerning microbiological monitoring of the products and production environment in the year 2018 and January 2019. Further results from official sampling performed according to the ordinary national sampling schedule by the DVFA during the year 2018 and January 2019 were included in this investigation to establish the possibility of the product being the source of the outbreak.

### Outbreak investigation timeline

In order to examine the efficiency of the outbreak investigation, we described the time between events of the investigation. We calculated the mean time from stool collection date to receival of the isolate at the SSI laboratory and the time to a case was linked to the outbreak by WGS.

## Results

### Cluster analysis

**Fig. 1.**
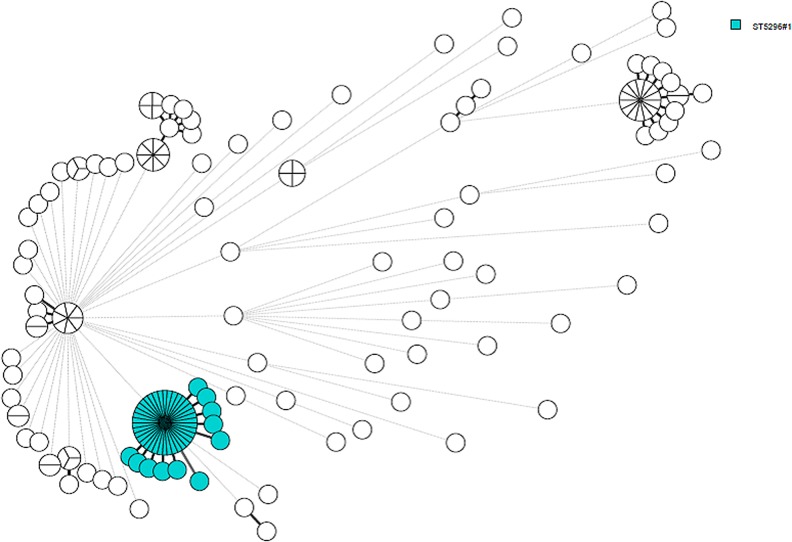
A minimum spanning tree calculated on cgMLST in BioNumerics. All human ST34 isolated in Denmark between August 2018 and March 2019 are included in the tree where the outbreak cluster is marked in blue. The branch lengths correspond to allelic differences and the thick branches represent one, two or three differences. Other genetic clusters were identified in this time period as seen on the figure.

The outbreak strain was ST5296 (aroC 866, dnaN 19, hemD 12, hisD 9, purE 5, sucA 9 and thrA 2) which is a single locus variant to the typical *S*. Typhimurium monophasic variant ST34. Within the genetic cluster ST5296 cluster 1 there were 0–3 allelic differences and a minimum of eight allelic differences to the nearest neighbours which were all ST34, Figure 1.

### Descriptive epidemiology and hypothesis

**Fig. 2.**
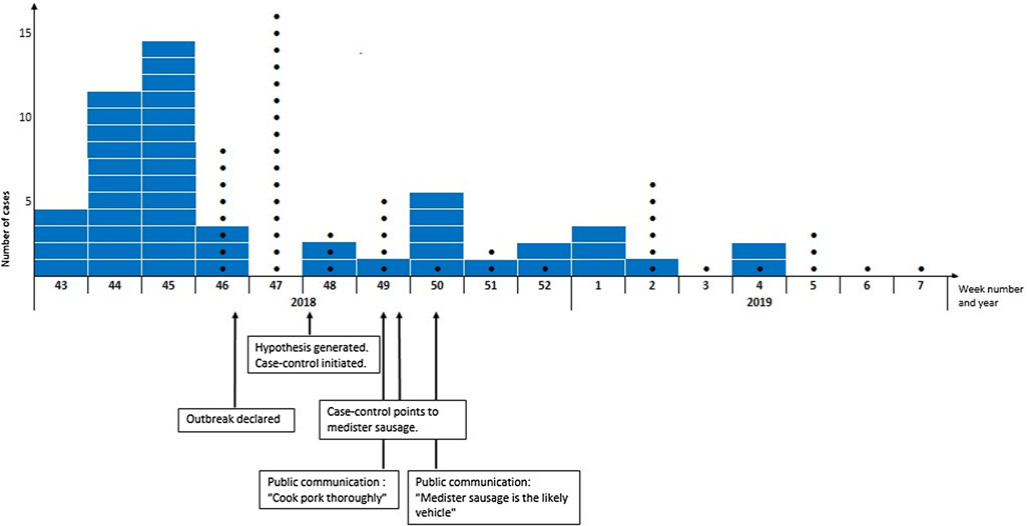
Timeline of the investigation of the outbreak of monophasic *S.* Typhimurium ST5296 cluster 1, Denmark October 2018 to January 2019, *n* = 49. In blue are number of cases per week according to stool collection date. Black dots are the number of cases per week that were linked to the outbreak by whole genome sequencing.

In total, 49 patients fulfilled the case definition. The median age was 65 years (range 11 months–97 years) and 53% were male. In total, 14% of cases (*n* = 7/49) were children ⩽18 years of age. Patients were geographically distributed across Denmark. In total, 43 interviews with patients were conducted. Of those, 28 were hypothesis-generating interviews and 15 were case-control study interviews. Sixty-one percent (30/49) were hospitalized and 35% (13/37) reported bloody diarrhoea as part of their symptoms. There were no deaths. The onset date of illness was known for 38 of the 49 patients and was between 14 October 2018 and 17 January 2019. According to the stool collection date known for all 49 cases, the outbreak lasted from week 43, 2018 to week 4, 2019 and peaked in weeks 43–45, Figure 2.

**Fig. 3.**
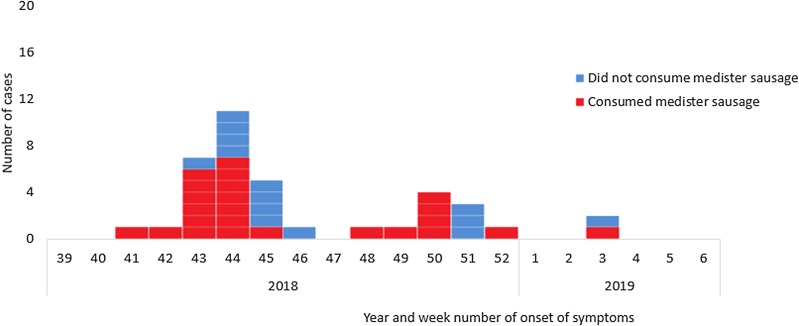
Consumption of medister sausage by year and week of onset of symptoms for cases of monophasic *S.* Typhimurium ST5296 cluster 1 with a known onset of disease (*n* = 38/49), Denmark October 2018 to January 2019.

None of the patients were linked socially and had not participated in any common events. Ninety-three percent (40/43) of interviewed patients had eaten fresh pork and 65% (28/43) had eaten medister sausage in the week prior to becoming ill with salmonella, Figure 3. Six patients mentioned that they had partly eaten the medister sausage raw (tasted the meat) or undercooked. Five patients who had eaten medister sausage informed that they had failed to boil the sausage prior to frying as is normally recommended on the packages and by the DVFA. The main shopping site was supermarket chain A (*n* = 19). Four cases that occurred late in the outbreak (stool collection date in December or January) and who had eaten medister sausage, mentioned that they had either bought the sausage frozen or had kept the sausage in the freezer at home.

Information from the initial eight hypothesis generating interviews conducted in November 2018 formed the rationale for a case-control study. Here seven patients mentioned that they had eaten medister sausage, which was higher than expected. Two families with ill children mentioned suspecting that the sausage was causing the illness. In one family the child had eaten medister sausage that was not thoroughly cooked. In the other family both the child and another family member became ill after sharing the sausage, although only the child was tested for salmonella. Several patients mentioned that they had bought the sausage on sale at a particular supermarket chain A.

### Case-control study

Twenty-one cases and 67 controls were included in the case-control study and interviewed between 28 November and 4 December 2018. For the majority of cases in the case-control study (*n* = 13), three controls were included. For two cases it was only possible to include one and two controls, respectively. For five cases, four controls were included and for one case five controls were included. For six cases, data from the hypothesis-generating interviews were included in the case-control study without re-interviewing the cases. The median age was 66 years (range 11 months–86 years) and 59% were male. In the univariable analyses, medister sausage, pork chops and mixed ground veal and pork showed a significant association with illness, Table 1. The mOR of medister sausage was 26.3 (95% CI 3.3–207) and explained 62% of cases. In a multivariable analysis, only medister sausage remained significant (mOR 51 (95% CI 3.4–770)). However, pork chops were borderline significant (mOR 8.7 (95% CI 1.0–77)). No supermarket chain was significantly associated with disease, but 76% of cases compared to 60% of controls had bought food in supermarket chain A (univariable mOR 2.3, 95% CI 0.7–7.3).

**Table 1.** Number and proportion of case and controls exposed and mOR from univariable analyses, by exposure, Denmark 2018

	Cases (*N* = 21)		Controls (*N* = 67)			
Exposure	Exposed	% Exposed	Exposed	% Exposed	mOR	95% CI
Food items						
Chicken	14	67	35	52	1.8	0.6–5.0
Ground beef	17	81	43	64	3.1	0.8–11.8
Other beef	9	43	29	43	0.9	0.3–2.6
Salami	9	43	29	43	0.9	0.3–2.4
Liver pate	13	62	35	52	1.5	0.5–4.1
Pork chops	8	38	11	16	3.7	1.1–12.8
Ground veal and pork	13	62	21	31	3.5	1.3–9.8
Ground pork	6	29	16	24	1.4	0.5–4.5
Medister sausage	13	62	11	16	26.3	3.3–206.9
Supermarket chains^a^						
A	16	76	40	60	2.3	0.7–7.3
B	7	33	18	27	1.4	0.5–4.1
C	5	24	17	25	1.0	0.3–3.2
D	9	43	21	31	2.2	0.6–7.2
E	11	52	32	48	1.2	0.4–3.2
F	5	23	18	27	1.0	0.3–2.9

a Included in the table are supermarket chains were more than 25% of the interviewed had shopped in the 14 days prior (to disease for cases).

### Analyses of food products

None of the 582 analysed isolates sequenced by DVFA in the National salmonella surveillance from animals, food and feed in 2017 to 2019 were designated to be ST5296 nor to be closely related (<10 SNPs) to the human outbreak isolates.

Relevant samples were only available from one case. Two separate packages of medister sausage were obtained for analyses. These sausages had been purchased at the same time as the sausage consumed by the case before becoming ill with salmonella. Both sausages were sampled and analysed (5 × 25 g samples). Salmonella was not detected in any of the 10 samples.

### Consumer purchase data investigation and trace-back investigation

In total 17 patients, who had eaten medister sausage, were contacted and invited to provide purchase data. Of those three patients mentioned that they only used cash and had not kept the receipts, one case could not access web-bank statements and seven patients failed to provide the requested information despite reminders by email. In total, six cases provided data from web-bank statements identifying purchases that they had made in supermarkets the 2 weeks before date of onset of illness or from the specific date they had bought medister sausage. For two of these cases, the purchase data returned from the supermarket chain did not include medister sausage. Information from three of the six cases showed that medister sausage was bought in supermarkets belonging to the same supermarket chain A. The last of the six cases had bought medister sausage from another supermarket chain. This medister sausage was a private label product produced by more than one manufacturer for this supermarket chain. This medister sausage could have been manufactured by the same manufacturer supplying supermarket chain A.

The trace-back investigations based on information from the hypothesis-generating and case-control interviews as well as the consumer purchase data investigation, pointed to one specific manufacturer of minced meat and prepared meat products (Manufacturer A). The manufacturer's own-check records concerning microbiological monitoring of the products and production environment in the year 2018 and January 2019 were investigated by the DVFA. At least one batch of minced meat and prepared meat were routinely sampled every week (five samples for each batch). In total, 90 batches of minced meat and 90 batches of prepared meat (whereof nine were batches of medister sausage) were analysed for salmonella. Only one sample from a batch of minced pork patty sampled in January 2019 showed the presence of salmonella (not related to the outbreak strain). No batches of medister sausage were positive for salmonella.

The DVFA also investigated the results of routine sampling performed at the slaughterhouse providing meat to Manufacturer A. As part of the mandatory sampling, one out of every 1000 carcasses were swap sampled and analysed for salmonella. Salmonella was detected eight times in these swap samples, but none were identical to the outbreak strain.

In the above mentioned time period, the DVFA food inspection units and meat inspection personnel took verification samples routinely at both the slaughter level (weekly) and at Manufacturer A. These results showed that only two samples were salmonella positive in the period investigated.

Manufacturer A took environmental swap samples from equipment and product bearing surfaces weekly and analysed for total bacterial growth as part of their normal surveillance scheme – none of these samples showed excessive counts. The cleaning and disinfection was visibly inspected daily by the staff. No breaches in procedures or obvious incidents that could explain the presence of a specific type of salmonella in multiple batches of medister sausage in a prolonged period of several weeks were identified. No interventions at the production site were deemed necessary by the DVFA.

Because the shelf life of medister sausage is short and fresh meat is used for the production, none of the actual batches of raw material meat used in the production of medister sausages or medister sausage batches suspected of having caused illness were available from Manufacturer A at the time the outbreak was detected and investigation initiated.

The medister sausages produced by Manufacturer A were packed at the production site and no handling of the actual sausage took place at the supermarket level.

### Public health actions

On 5 December 2018, with 28 confirmed cases in the outbreak, a news item was issued informing the public about the outbreak and that it was likely to be caused by pork. The public was advised to thoroughly cook pork before consumption and to avoid tasting or eating raw or undercooked pork. On 12 December 2018, information to the public was published on the SSI website that a likely source of the outbreak was medister sausage possibly together with other pork products. The news item included a recommendation to both boil and fry sausages like medister sausage before consumption.

### Outbreak investigation timeline

A timeline with an overview of the steps in the outbreak investigation is shown in Figure 2. The mean time from stool collection date to a case was linked to the outbreak by WGS was 20 days (range 14–29 days). The mean time from stool collection date to receival of the isolate at the SSI laboratory was 7 days (range 3–19 days) and the mean time from the isolate had been received at the SSI laboratory and a case was linked to the outbreak by WGS was 13 days (range 9–23 days).

## Discussion

From our investigations we conclude that the most likely vehicle for the majority of cases in this outbreak of monophasic *S.* Typhimurium comprising 49 patients was medister sausage. However, since not all cases had consumed medister sausage we also conclude that other pork products might have been involved. Medister sausage is a traditional Danish product that is often served in the late autumn months and Christmas holiday season. It is a long, thick sausage made from ground pork, seasoned and stuffed into casings. In contrast to other sausages on the Danish market, medister sausage is a raw meat product. The official recommendation is to first boil the sausage before frying it. This should be done to ensure sufficient heat treatment before consumption as the centre of the sausage might still be raw if it is only fried. In theory, some of the outbreak cases that did not consume medister sausage, could also be due to cross-contamination in case a family member had bought and handled a medister sausage. Cases were not interviewed systematically on this issue and we recommend to consider this is in future outbreak investigations.

We could not confirm by the tracing and the microbiological investigations that medister sausage from Manufacturer A was the cause of part of the outbreak. However, although this particular ST of salmonella had not been detected before, it was closely related to the type often associated with pork, ST34, differing with variation in the MLST gene *aroC*. No isolates from the surveillance of food and feed in 2018 and 2019 matched the outbreak strain and salmonella was not detected in any of the samples of medister sausage that were available. However, it is likely that the bacteria might be present in low numbers and unevenly distributed in the raw material and products and the chance of detecting it at sampling thus might be limited. It is also likely that only a low salmonella dose is sufficient for patients to become ill as medister sausage is a product with a high content of fat (10–20%) that protects the bacteria past the barrier of the gastric acid. This has been hypothesized in previous outbreaks of salmonella involving fatty products like cheese and chocolate [11, 12]. Although, several cases in the outbreak exhibited risk behaviour as they expressed deliberately tasting raw sausage or consuming the product in an undercooked state, the high fat content might also increase the thermal resistance of the bacteria so that it is not fully eliminated by heating [12]. According to European Union legislation, salmonella may not be detected in samples of ground meat, prepared meat (e.g. raw sausages, marinated meat) and ready to eat meat products whereas it is allowed in fresh meat. That salmonella can accidentally be present in medister sausage is, however, highlighted by the fact that samples of medister sausage from more manufacturers, as part of routine surveillance and establishment own check analyses, were found positive for salmonella during the period of the outbreak. This includes the finding of the serovars Mbandaka, Typhimurium and its monophasic variant (different from the outbreak strain).

The outbreak peaked in late October to early November, 2018. Several late cases had mentioned in interviews having eaten medister that had been frozen which could explain the occurrence of these late cases. However, cases were not interviewed systematically concerning freezing of medister.

Outbreaks of *S.* Typhimurium associated with several pork products including risk-products have previously been detected in Denmark several times. In 2010, in an outbreak lasting 8 months causing illness in 172 registered patients, a number of different pork products caused illness, including a ready-to-eat spreadable pork sausage ‘*Tee-wurst*’ also to be viewed as a risk-product as the production does not include a thorough heat treatment step [13]. In 2008, several pork products from Denmark caused an outbreak of *S.* Typhimurium in Denmark, Norway and Sweden [14]. This outbreak was caused by several different pork products including medister sausage and 4 out of 37 cases died. We speculated at the time, that contamination of medister sausage could lead to high ingestion doses of salmonella and thus to particularly serious infections [14]. A relatively high proportion of cases was hospitalized in the current outbreak (61%) and 35% reported bloody diarrhoea; this compares to four previous outbreaks of monophasic *S.* Typhimurium in Denmark where between 15% and 35% of patients reported being hospitalized [11–13 15].

Previous outbreaks with known transmission from several different types of pork products originated from single slaughterhouses where contamination at the slaughterhouse likely caused contamination of several different pork products [13, 14]. In the current outbreak, we did not have sufficient information allowing us to establish the mode of contamination and there was no straightforward explanation as to why cases were becoming ill over a time period exceeding the period of a single production batch of medister sausage and thus likely including more than one batch of sausage. This highlights, that even though the Danish production of pork is under a strict salmonella control program, there is still a lot to be understood about the possible routes and mechanisms of contamination and persistence of salmonella in the products. The contamination is expected to have been limited to the slaughterhouse level in combination with the production level either as a series of limited events or as a result of a persistent contamination for a shorter period of time at the slaughterhouse or manufacturing level. Even though the current investigation did not manage to fully reveal the mechanism of contamination of the meat, continuing to do thorough outbreak investigations in the future will hopefully help in elaborating the knowledge on possible transmission routes in relation to the production facilities.

We applied the use of consumer purchase data in order to examine whether a specific brand of medister sausage was the cause of the outbreak. This method has been highly effective in solving foodborne outbreaks in Denmark before, both for identifying a specific product [16] and as a hypothesis generating tool [17 18]. The consumer purchase data method has also been used with success internationally [10]. In this outbreak investigation we only received purchase data from 6/17 patients and only for four cases did the data include information on medister sausage and this part of the investigation did not lead to an identification of a specific brand or production batch or period of medister sausage.

In the case-control study, we included both cases interviewed with the hypothesis-generating questionnaire in addition to the cases interviewed with the specifically designed case-control questionnaire. Since the case-control questionnaire was adapted from the hypothesis-generating questionnaire and questions were asked in the same way, we did not believe that this would lead to bias in our results. We did, however, also perform the analyses excluding the six cases who were only interviewed using the hypothesis generating questionnaire and their matched controls (*n* = 16). In the resulting univariable analyses, only medister sausage was found to be significantly associated with illness. We asked controls about medister sausage consumption 2 weeks prior to the interviews, which was a slightly later period than the onset date of illness for cases. Because medister sausage consumption is seasonal, this approach could bias the results. However, this bias would likely results in a lower OR because consumption of medister sausage in Denmark generally increases towards Christmas.

We took the opportunity to investigate the timeliness of the outbreak investigation and found a mean time of 20 days from the faecal sample was taken to a case was linked to the outbreak by WGS. This time included the sample being received and analysed at the initial laboratory, time to receival of the isolate at the SSI and handling of the isolate including WGS. WGS has become an important tool in outbreak investigations, but has not reduced the time to case ascertainment in salmonella investigations. However, since the outbreak occurred steps have been taken to reduce this time. A designated transportation service that picks up isolates directly at the primary laboratories every morning and delivers the isolates directly to the SSI, has been introduced. Furthermore, WGS was previously run twice a week, but this has been increased to three times a week. These steps should have improved the possibility of handling outbreaks in a timelier manner.

In conclusion, in spite of having eliminated salmonella in the egg and broiler industry, Denmark is still at risk of major outbreaks involving other food sectors including the pig industry. Consumers have to make sure that pork is handled correctly, in particular when it comes to raw products that need to be thoroughly cooked before consumption. Tasting raw meat or eating undercooked pork meat should be discouraged.

## References

[1] Anonymous (2018) Annual Report on Zoonosis in Denmark 2017. National Food Institute, Technical University of Denmark.

[2] Statens Serum Institut. Overvågning i tal, grafer og kort n.d. Available at https://statistik.ssi.dk/sygdomsdata (Accessed 13 May 2019).

[3] Ethelberg S Uge 15–2018 n.d. Available at https://www.ssi.dk/aktuelt/nyhedsbreve/epi-nyt/2018/uge-15---2018 (Accessed 13 May 2019).

[4] Wegener HC (2003) Salmonella control programs in Denmark. Emerging Infectious Diseases 9, 774–780. doi: 10.3201/eid0907.030024. 12890316 PMC3023435

[5] Anonymous (2017) Annual Report on Zoonoses in Denmark 2016. National Food Institute, Technical University of Denmark.

[6] Anonymous (2016) Annual report in Zoonoses in Denmark 2015. National Food Institute, Technical University of Denmark.

[7] Anonymous (2015) Annual Report on Zoonoses in Denmark 2014. National Food Institute, Technical University of Denmark.

[8] Pedersen CB (2006) The danish civil registration system. A cohort of eight million persons. Danish Medical Bulletin 53, 441–449. 17150149

[9] Kaas RS (2014) Solving the problem of comparing whole bacterial genomes across different sequencing platforms. PLoS ONE 9, e104984. doi: 10.1371/journal.pone.0104984. 25110940 PMC4128722

[10] Møller FT, Mølbak K and Ethelberg S (2018) Analysis of consumer food purchase data used for outbreak investigations, a review. Eurosurveillance 23, pii=1700503. doi: 10.2807/1560-7917.ES.2018.23.24.1700503. PMC6152197 29921346

[11] Van Duynhoven YTHP (2009) A prolonged outbreak of *Salmonella* Typhimurium infection related to an uncommon vehicle: hard cheese made from raw milk. Epidemiology & Infection 137, 1548–1557. 19296867 10.1017/S0950268809002337

[12] Werber D (2005) International outbreak of Salmonella Oranienburg due to German chocolate. BMC Infectious Diseases 5, 7. 15691371 10.1186/1471-2334-5-7 PMC552305

[13] Kuhn KG (2013) A long-lasting outbreak of *Salmonella* Typhimurium U323 associated with several pork products, Denmark, 2010. Epidemiology & Infection 141, 260–268. 22717213 10.1017/S0950268812000702 PMC9152058

[14] Bruun T (2009) An outbreak of *Salmonella* Typhimurium infections in Denmark, Norway and Sweden, 2008. Eurosurveillance 14, pii: 19147. 19317986

[15] Kuhn K (2011) An outbreak of *Salmonella* Typhimurium traced back to salami, Denmark, April to June 2010. Eurosurveillance 16, pii: 19863. 21596006

[16] Gillesberg Lassen S (2013) Ongoing multi-strain food-borne hepatitis A outbreak with frozen berries as suspected vehicle: four Nordic countries affected, October 2012 to April 2013. Eurosurveillance 18, 20467. 23647625

[17] Ethelberg S (2009) Outbreak of non-O157 Shiga toxin-producing Escherichia coli infection from consumption of beef sausage. Clinical Infectious Diseases 48, e78–e81. 19272017 10.1086/597502

[18] Müller L (2016) Outbreak of *Salmonella* strathcona caused by datterino tomatoes, Denmark, 2011. Epidemiology & Infection 144, 2802–2811. 26846608 10.1017/S0950268816000121 PMC9150450

